# Preparation of Portland Cement-Free Autoclaved Aerated Concrete Using Yellow River Sediment

**DOI:** 10.3390/ma19091820

**Published:** 2026-04-29

**Authors:** Huawei Shi, Xiaosheng Zhou, Ge Zhang, Kunpeng Li, Chen Chen, Zekun Dong, Jialing Li

**Affiliations:** 1 Yellow River Institute of Hydraulic Research, Yellow River Water Conservancy Commission, Zhengzhou 450003, China; 15538352232@163.com (H.S.); gezhangyrihr@163.com (G.Z.); 15617633649@163.com (C.C.); zekundong@126.com (Z.D.); jialingli1120@163.com (J.L.); 2 Henan Provincial Engineering Research Center of Reservoir-Lake Function Restoring and Maintaining, Zhengzhou 450003, China; 3 Key Laboratory of Yellow River, MWR, Zhengzhou 450003, China; 4 Ningxia Hui Autonomous Region River and Lake Affairs Center, Yinchuan 750002, China

**Keywords:** AAC samples, Yellow River sediment, compressive strength, bulk density, microstructure

## Abstract

**Highlights:**

**What are the main findings?**

- Yellow River sediment was valorized to fabricate cement-free autoclaved aerated concrete (AAC) with balanced density and mechanical strength.
- The water-to-material ratio and quartz sand substitution were identified as key factors governing the pore structure and formation of a robust tobermorite–C-S-H gel matrix.

**What are the implications of the main findings?**

- High-value utilization of Yellow River sediment with eco-economic benefits can be realized. This work provides technical reference for low-cement, high-solid-waste AAC development.
- The revealed correlation mechanism between “macroscopic properties—microstructure—characteristic products” of sediment-based AAC provides theoretical support for the application of similar solid wastes in the construction materials field.

**Abstract:**

To solve quartz sand shortage and poor mechanical properties of fly ash-based autoclaved aerated concrete (AAC) in traditional production, this study prepared AAC blocks using Yellow River sediment as the main siliceous raw material, combined with slag, quicklime and other additives. Seven sample groups (S1–S7) with different mix proportions were designed by adjusting the water-to-material ratio and replacing some raw materials with quartz sand or fly ash. The results showed that as the water-to-material ratio increased (S1–S4), AAC slurry fluidity improved, but foaming rate and expansion volume showed a non-monotonic trend. With the same water-to-material ratio, the S5 sample had lower bulk density (695 kg/m^3^) and moderate but favorable strength (4.25 MPa). Microscopic analysis revealed AAC strength mainly derived from tobermorite and C-S-H gel, and xonotlite enhanced structural stability. This study provides a feasible method for resource utilization of Yellow River sediment in AAC production, with environmental and engineering value.

## 1. Introduction

Autoclaved aerated concrete (AAC) possesses a well-developed pore structure, which endows it with superior lightweight properties and excellent thermal insulation performance. At present, it enjoys extensive applications across the construction domain [1,2,3,4]. This versatile material can be employed in building envelope structures, including floors, walls, and roofs. Moreover, it plays a crucial role in interior partitions and decoration work within buildings. The lightweight property of autoclaved aerated concrete effectively reduces the load borne by buildings, thereby significantly enhancing building safety. Simultaneously, its remarkable heat insulation advantages contribute to substantial energy savings and a notable reduction in carbon emissions, making it an environmentally friendly and energy efficient choice in modern construction [5].

In the traditional material system of autoclaved aerated concrete, lime, quartz sand, fly ash and similar materials are extensively utilized [6]. However, as a result of the large-scale consumption of resources, quartz sand has been in a state of severe shortage, with demand far outstripping supply. When it comes to manufacturing autoclaved aerated concrete using fly ash, the mechanical properties of the resulting products tend to be rather subpar [7]. In view of these problems, researchers launched a host of in-depth studies on leveraging various industrial solid wastes, such as iron tailings, slag, and red mud, to improve the performance of aerated concrete [8,9,10,11]. Kittipong et al. [12] incorporated rice-husk ash (RHA) and aluminum (Al)-containing waste (AW) as partial aggregate replacements in the production of autoclaved aerated concrete, which not only reduces the amount of waste discharged into the environment but also shortens the autoclaving curing time or lowers the autoclaving curing temperature. Muhammad et al. [13] used waste granite dust as a partial replacement for sand in autoclaved aerated concrete (AAC), which can reduce its hazards to human health and the environment while meeting the demand for sand and alleviating the problem of sand resource depletion. Their efforts have made remarkable contributions to the secondary utilization of solid wastes, not only promoting the sustainable development of the construction materials industry but also effectively alleviating the pressure on natural resources and environmental pollution.

Pachideh et al. [14] probed how pozzolanic materials including silica fume, zeolite and granulated blast-furnace slag act on the performance of aerated concrete. Their experimental findings demonstrated that the incorporation of such pozzolanic materials could notably enhance the mechanical properties of aerated concrete. Specifically, when the content of pozzolanic materials reached 21% of the cement mass, the tensile strength of the concrete was elevated by 25%, and a marked decline was observed in its water absorption rate. Zhang et al. [15] used dredged river sediment to prepare AAC blocks. The results revealed that bricks produced with 30–34% dredged river sediment and other components under specified conditions boasted a 4.5 MPa compressive strength and 716.56 kg/m^3^ density, complying with national standards. However, the dosage of sediment was relatively low, with the maximum sediment content only accounting for 42%, while a large amount of cement was used (the maximum cement content reached 48%), which is inconsistent with the concept of low-carbon environmental protection. Therefore, how to further increase the dosage of dredged sediment and reduce the cement content on the premise of ensuring that key indicators such as mechanical properties and durability of aerated concrete meet service requirements, so as to realize the synergistic development of high-value utilization of dredged waste and low-carbon building materials, has become a key technical issue that urgently needs to be solved in the current fields of water conservancy engineering waste recycling and greening of building materials.

The sedimentation problem of the Yellow River is the most serious in the world. The massive accumulation of sediment not only occupies a large number of land resources but also poses a serious threat to the safety of water conservancy projects and river basin ecological environment. The main components of Yellow River sediment are SiO_2_, Al_2_O_3_, CaO, and K_2_O. Among them, the content of SiO_2_ and Al_2_O_3_ accounts for about 70% to 80%, which is similar to quartz sand. It can be used to prepare bricks, ceramsite and other building materials. The strength of autoclaved aerated concrete mainly stems from the cementitious substances produced by the alkali activator and slag. Notably, the raw materials contain no cement. Meanwhile, the alkali activator can stimulate the Yellow River sediment to participate in the hydration gel reaction. Under the effect of the alkali activator, the siloxane bonds and aluminate bonds in the Yellow River sediment depolymerize and undergo polycondensation with the active components in the system, generating hydration products with a three-dimensional network structure and thus endowing the material with mechanical strength [16]. The Yellow River sediment is characterized by fine particle size and large specific surface area, which contributes to a more efficient alkali activation. This approach can effectively mitigate the adverse impact of fine-grained sediment on concrete performance.

The overall research objectives of this study were to realize the high-value utilization of Yellow River sediment by preparing Portland cement-free AAC with Yellow River sediment as the main siliceous raw material and to reveal the regulation mechanism of material composition and preparation process on the performance of sediment-based AAC so as to provide a new technical approach for the resource utilization of Yellow River sediment and the development of low-carbon AAC. In addition, this material has a lower production cost than traditional cement-based AAC, rendering it more advantageous in actual production and application.

## 2. Materials and Methods

### 2.1. Materials

**Figure 1:**
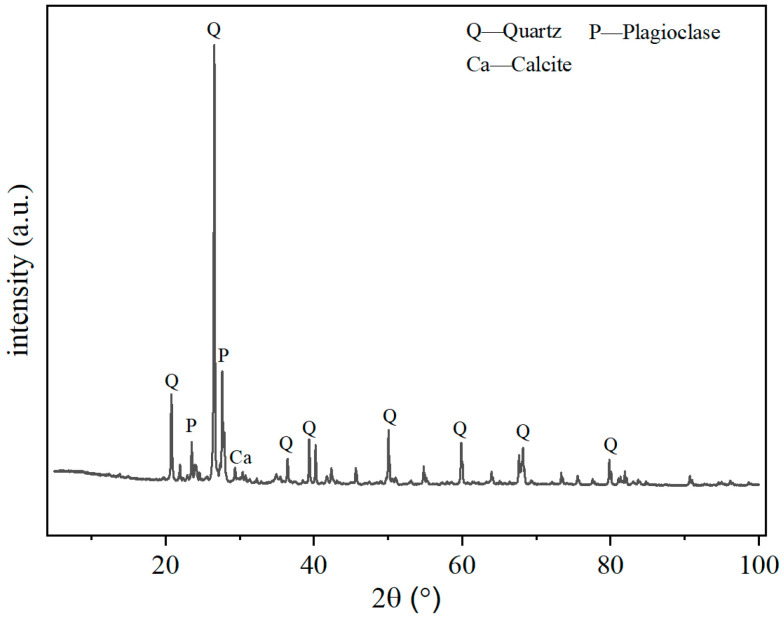
XRD pattern of Yellow River sediment.

The sediment employed in this study was collected from the Xixiayuan Reservoir section of the Yellow River. X-ray diffraction (XRD) analysis was conducted to identify its primary mineral constituents, which were found to be quartz, plagioclase, and calcium carbonate (Figure 1). Quantitative analysis of the mineral contents was performed using the Rietveld method, and the contents of quartz, plagioclase, and calcium carbonate were determined to be 63.18 wt%, 28.27 wt%, and 9.46 wt%, respectively. The median particle size of Xixiayuan Reservoir sediment particles was about 0.08 mm. The sediment was sieved through a 200-mesh sieve to remove the coarse-grained particle components.

**Figure 2:**
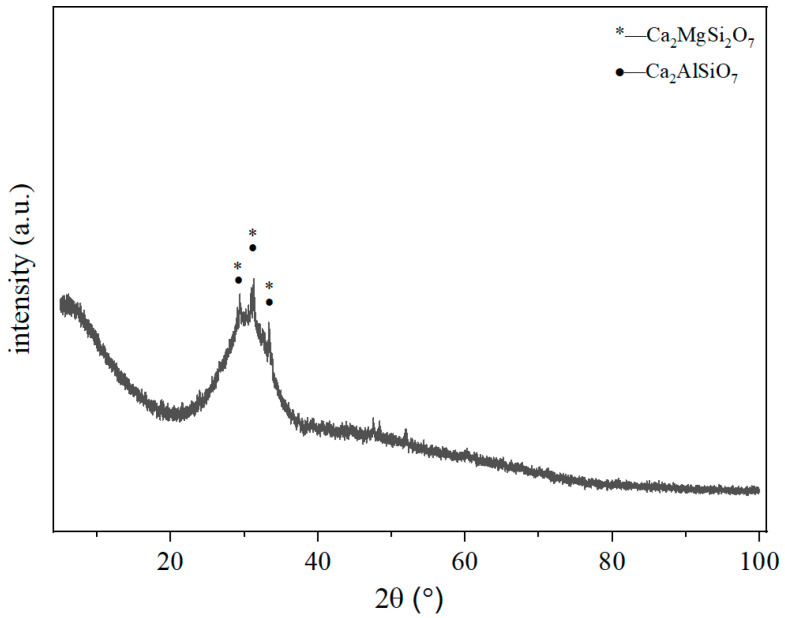
XRD pattern of S95 grade slag powder.

The slag powder adopted for the present test was of S95 grade, purchased from Qianhao Mineral Products Processing Plant, Lingshou County, Shijiazhuang City, Hebei Province. The median particle size of the slag powder is approximately 12.38 μm. The mineral composition analysis of slag is shown in Figure 2. There is a large “steamed bun peak” between the diffraction angle of 20° and 40°, and there is also very little melilite phase. Melilite is a solid solution of calcium aluminum melilite (Ca_2_Al_2_SiO_7_) and calcium magnesium melilite (Ca_2_MgSi_2_O_7_). Both substances exist primarily in an amorphous state, making the slag highly susceptible to activation. The quicklime used in this study has a CaO content higher than 99% and was purchased from Beihai Jinghong Trading Co., Ltd. (Beihai, China). The median particle size of aluminum powder is 0.017 mm, was obtained from Jinan Jingsheng Chemical Co., Ltd., Jinan, China. The chemical compositions were tested by EDX, and the test results of various raw materials are shown in Table 1. The admixtures include foam-stabilizing agents, etc. Their function is to generate a more uniform pore structure.

The mix proportions of different samples are shown in Table 2. The aluminum powder dosage for all samples is 0.9 g. TEA stands for Triethanolamine, which acts as a foam stabilizer to stabilize the pore structure. Sample 1 to Sample 4 are designed to investigate the changes in strength and dry density of autoclaved aerated concrete blocks under different water consumption conditions, while Sample 5 to Sample 7 are intended to explore the performance changes of autoclaved aerated concrete blocks under the influence of quartz sand and fly ash. The Ca/Si ratio refers to the molar ratio of total calcium oxide (CaO) to total silicon dioxide (SiO_2_) in the raw materials. The Ca/Si ratios of different samples S1–S4, S5, S6, and S7 are 0.68, 0.50, 0.72, and 0.72 respectively.

### 2.2. Methods of Preparation and Detection

**Figure 3:**
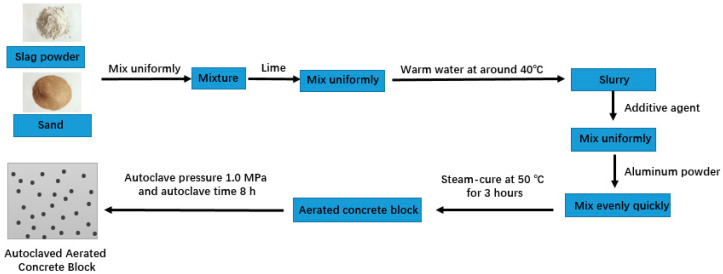
Preparation process of Yellow River sediment AAC blocks.

The preparation process of AAC blocks is illustrated in Figure 3. The sediment from the Yellow River and the slag powder were weighed according to the mix proportion and agitated evenly. Then a certain amount of quicklime was added and blended thoroughly. Warm water was added in line with the mix proportion and blended until uniform. Next, the admixtures were added and blended evenly, and the mixture was allowed to stand for 2 min. While stirring, aluminum powder was added to the slurry until a homogeneous slurry was formed. The slurry was poured into the sample mold, which was then placed into a steam curing box at 45–50 °C for 3 h. During this period, the slurry foamed into a sample with a stable volume. Finally, the sample was placed in an autoclave and cured at 180 °C and 1.0 MPa for 8 h. The autoclaved aerated concrete was obtained by removing the block after the autoclave cooled to room temperature.

### 2.3. Testing Methods

#### 2.3.1. Fluidity of Slurry

The fluidity of autoclaved aerated concrete slurry is a key factor affecting its molding quality and final performance. The fluidity of slurry was tested using a cylindrical mold (inner diameter = 50 mm and height = 100 mm). First, the mixture was poured into the cylindrical mold positioned on a glass plate, which was then lifted rapidly. Subsequently, a ruler was employed to measure the diameters of the slurry in two perpendicular directions, and the average value was taken as the slurry fluidity.

#### 2.3.2. Gas Foaming Rate

To measure the expansion volume, 100 mL of slurry was first poured into a 250 mL measuring cylinder, which was immediately transferred to a 50 °C curing chamber. Next, the volume of the foaming slurry was recorded at 2 min intervals, and the measurement was terminated once the volume ceased to change.

#### 2.3.3. Bulk Density

The bulk density of the samples was determined after drying and curing, in accordance with the national standard GB/T 11968-2020. Three parallel samples were dried in an oven at 65 ± 5 °C for 24 h, then further dried at 85 ± 5 °C for another 24 h, and finally dried at 105 ± 5 °C until a constant mass was achieved. Then, the bulk density was calculated by the mass-volume method.

#### 2.3.4. Compressive Strength

The compressive strength of the specimens was determined via a microcomputer-controlled electro-hydraulic servo pressure tester. Three parallel specimens with dimensions of 100 mm× 100 mm× 100 mm were prepared for each group, and the tests were conducted at a loading rate of 5.0 kN/s.

#### 2.3.5. Air Pore Structure

A digital camera was used to take high-definition pictures of the cross-section of the block (100 × 100 mm). MATLAB R2024a software was employed to intercept the central area of 50 × 50 mm for binarization processing. In the processed image, air pores are displayed in black, and pore walls are shown in white. An algorithm was used to calculate information such as the number of air pores of different sizes, air pore area, and average air pore diameter.

#### 2.3.6. Microscopic Testing

The mineral composition of autoclaved aerated concrete was tested using a Rigaku Ultima IV X-ray diffractometer (XRD) (Rigaku Corporation, Tokyo, Japan) under the following conditions: 2θ range of 10–50°, scanning rate of 5°/min, and operating voltage and current of 40 kV and 100 mA, respectively. Fourier Transform Infrared (FTIR) spectroscopy was tested using a Shimadzu IRTracer 100 Fourier Transform Infrared Spectrometer (Shimadzu Corporation, Kyoto, Japan) with the following parameters: resolution of 4 cm^−1^, detector type of MIR TGS, and measurement range of 500–4000 cm^−1^. The TG-DTA was conducted by a ZCT-B simultaneous thermal analyzer (Beijing Jingyi Hitechinstrument Co., Ltd., Beijing, China). Microstructural observations of the samples were performed using a Carl Zeiss Sigma 300 field emission environmental scanning electron microscope (Oberkochen, Germany).

## 3. Results and Discussion

### 3.1. Fluidity and Gas Foaming Rate

**Figure 4:**
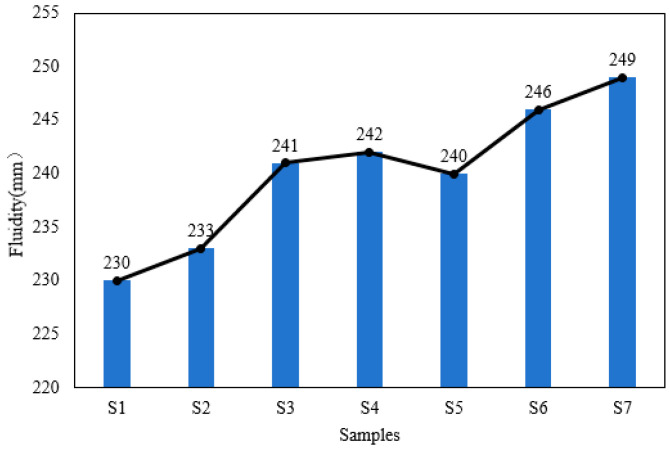
The fluidity of different AAC slurry samples.

The fluidity of the autoclaved aerated concrete (AAC) slurry for different samples is shown in Figure 4. As the water consumption increases, the fluidity of samples S1 to S4 gradually increases. For samples incorporating quartz sand and fly ash, their fluidity is higher than that of Sample S2. Specifically, the fluidity of samples where fly ash replaces slag exhibits a more significant upward trend. This phenomenon can be attributed to the fact that the water absorption capacity of quartz sand and fly ash is weaker than that of slag.

**Figure 5:**
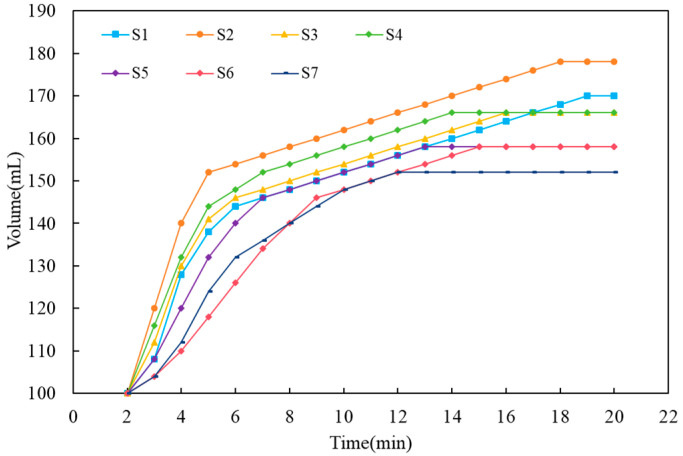
The gas-foaming rate of different AAC slurry samples.

The gas-foaming rate of autoclaved aerated concrete slurry for different samples is shown in Figure 5. As can be seen from the results, all curves share a similar changing pattern—starting with a sharp surge and then transitioning to a slower increment before leveling off. In the stage of rapid gas generation, the gas-foaming rate follows the descending sequence of S2 > S4 > S3 > S1 > S5 > S7 > S6. The final expansion volume in descending order is S2 > S1 > S4 > S3 > S5 > S6 > S7.

In general, the greater the fluidity of the slurry, the lower its viscosity. Under such circumstances, the external pressure exerted by the slurry on the pores formed by the gas foaming of aluminum powder is smaller, making it easier for the pores to grow. However, a comparison of samples S1 to S4 reveals that the gas-foaming rate and final gas expansion volume of the samples are not completely consistent with their fluidity, and Sample S2 exhibits the highest gas-foaming rate and final expansion volume. The formation of pores depends on two aspects. On the one hand, it relies on the gradual generation of hydrogen gas. On the other hand, it is determined by the pressure exerted by the slurry on the bubbles. The bubbles themselves exist in a pair of action and reaction forces formed by the slurry pressure, the gas pressure inside the bubbles, and the surface tension on the surface of the bubble walls. These forces acting on the bubbles are in a dynamically balanced state of interaction. This phenomenon occurs because as fluidity increases, large pores in the slurry may undergo pore coalescence, and the insufficient viscosity of the slurry causes some pores to escape. For samples S5 to S7, the increase in fluidity leads to the escape of more pores, thus resulting in relatively low gas-foaming rate and final expansion volume. This indicates that in addition to the dosage of aluminum powder, an appropriate water-to-material ratio is a key parameter affecting the pore formation of AAC.

### 3.2. Bulk Density and Compressive Strength

**Figure 6:**
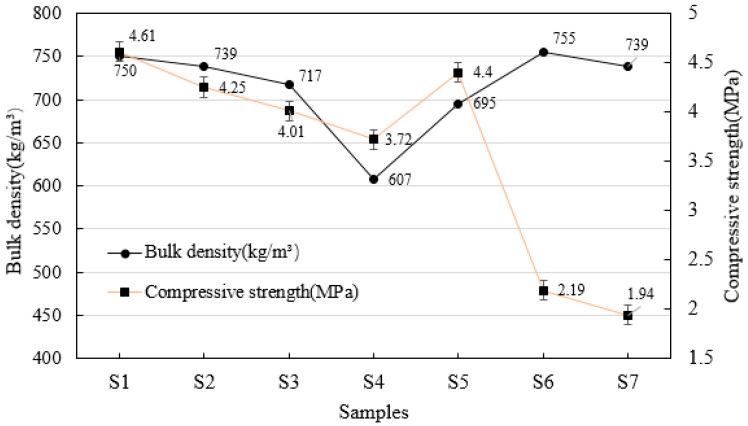
The bulk density and compressive strength of AAC samples.

The bulk density and compressive strength are key parameters for evaluating the properties of AAC, and there is a certain correlation between them. Generally, the lower the bulk density, the lower the compressive strength. In practical applications, we expect AAC blocks to have lower bulk density and higher compressive strength. Figure 6 shows the changes in bulk density and compressive strength of different AAC samples. With the increase in water-to-material ratio (S1–S4), the bulk density and compressive strength of AAC blocks decrease gradually. For the S5 sample where quartz sand is used to replace sediment, with the same water-to-material ratio, the S5 sample has lower bulk density and higher compressive strength than the S2 sample. This is because quartz sand has a higher SiO_2_ content, which promotes a more complete hydration reaction in the system, thereby resulting in higher strength of the corresponding blocks. For the S6 and S7 samples where fly ash is used to replace slag, the blocks exhibit higher bulk density but lower compressive strength, and cracks appear on the surface of the samples, which affects the strength of the blocks.

### 3.3. Air Pore Structure

Autoclaved aerated concrete (AAC) is a typical macroscopic porous material, and the classification characteristics of its pores directly affect the microstructure and properties of the aerated concrete. Pores larger than 2 mm are classified as macropores, those between 0.5 mm and 2 mm as mesopores, and those smaller than 0.5 mm as micropores. Many scholars have studied the effects of pore size distribution [17,18,19] and pore morphology [20,21,22,23] on strength. The general conclusion drawn is that the larger the pore structure and the higher the proportion of macropores, the lower the compressive strength of AAC blocks.

**Figure 7:**
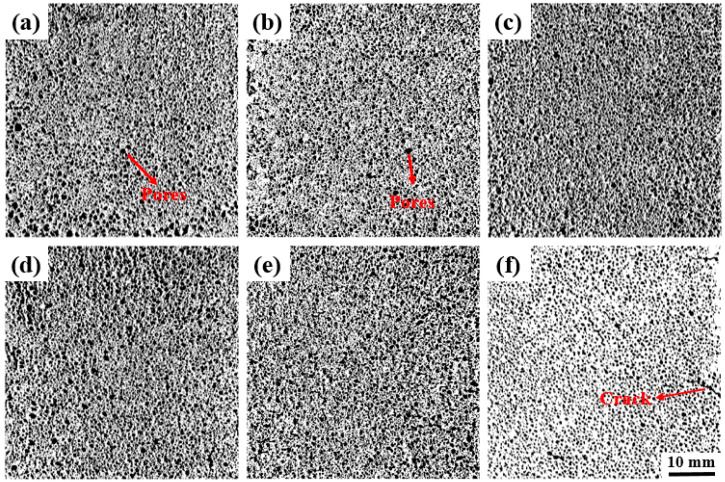
The air pore structure of AAC samples. (**a**) S1 sample, (**b**) S2 sample, (**c**) S3 sample, (**d**) S4 sample, (**e**) S5 sample, (**f**) S6 sample.

Figure 7 shows the surface pore structure diagrams of samples S1–S6 after binarization processing. Table 3 summarizes the relevant information on the pore characteristics of different samples. Overall, when the average pore diameter is smaller, the number and area proportion of macropores are lower, the number and area proportion of small pores and mesopores are higher, and the bulk density tends to be larger. For Samples S1–S4, as the water-to-material ratio increases, the fluidity of the slurry increases gradually, the proportion of macropores in the AAC blocks increases accordingly, and the bulk density and compressive strength of the AAC blocks decrease gradually. For example, Sample S6 has an average pore diameter of 0.3648 mm, only 1 macropore, and a macropore area of 22.62%, with a bulk density reaching 755 kg/m^3^, which is a relatively high level, while Sample S4 has an average pore diameter of 0.5532 mm, 12 macropores, and a macropore area of 64.45, with a bulk density of 607 kg/m^3^, which is relatively low. This indicates that a finer pore structure (with a smaller proportion of macropores, a larger proportion of small pores and mesopores, and a smaller average pore diameter) leads to a tighter filling of pores inside the material and a larger bulk density. Generally speaking, samples with a smaller average pore diameter, fewer macropores, and a lower area proportion of macropores have higher compressive strength. When in the pore structure, the role of macropores as stress concentration points is weakened (with fewer macropores and smaller area), and small pores and mesopores are distributed more uniformly, the material is less likely to be damaged due to stress concentration at macropores when subjected to force, and the compressive strength is improved. However, at the same time, factors such as bulk density also synergistically affect the compressive strength. If the bulk density is too low, even if the pore structure is good, the compressive strength may be limited. Although Sample S6 has a relatively large bulk density, there are cracks in its pore structure, which greatly affects the compressive strength.

### 3.4. Microanalysis

#### 3.4.1. XRD Analysis

**Figure 8:**
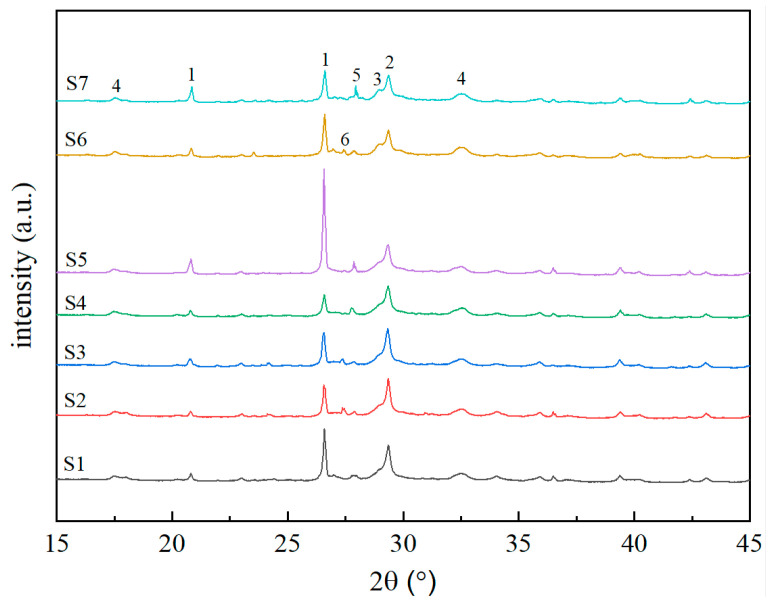
XRD patterns of AAC samples (1—quartz; 2—calcite; 3—tobermorite; 4—katoite; 5—anorthite; 6—xonotlite).

The mineral phases of autoclaved aerated concrete (AAC) samples were analyzed using X-ray diffraction (XRD). Figure 8 presents the XRD patterns of the AAC samples after autoclaved curing. All samples exhibit similar dominant mineral phases, including quartz, calcite, tobermorite, katoite, and anorthite, but significant differences in relative contents are observed due to varying mix proportions.

With the Ca/Si ratio maintained at 0.68, increasing the water-to-material ratio (S1–S4) progressively lowered the quartz content in AAC samples while enriching hydration products (tobermorite and katoite). Sample S5, however, had the maximum quartz content because its Ca/Si ratio dropped to 0.5 following the incorporation of quartz sand. The autoclaved curing process facilitated the reaction of siliceous and calcareous materials to produce highly crystalline tobermorite. This layered hydrate, combined with C-S-H gel, is responsible for the primary mechanical strength of the AAC blocks. It possesses high hardness and stability, enabling it to provide strong support and load-bearing capacity for the blocks [24,25]. Distinct xonotlite diffraction peaks are also observed in samples S2, S3, and S6. The formation of xonotlite can fill the internal pores of AAC blocks, rendering the pore wall structure more robust. Simultaneously, xonotlite forms an interwoven network with other hydration products (e.g., tobermorite), resulting in a more robust structural framework. This interconnected microstructure reinforces the continuity and wholeness of the matrix, which in turn contributes to the enhanced compressive strength of the AAC blocks.

#### 3.4.2. FTIR Analysis

**Figure 9:**
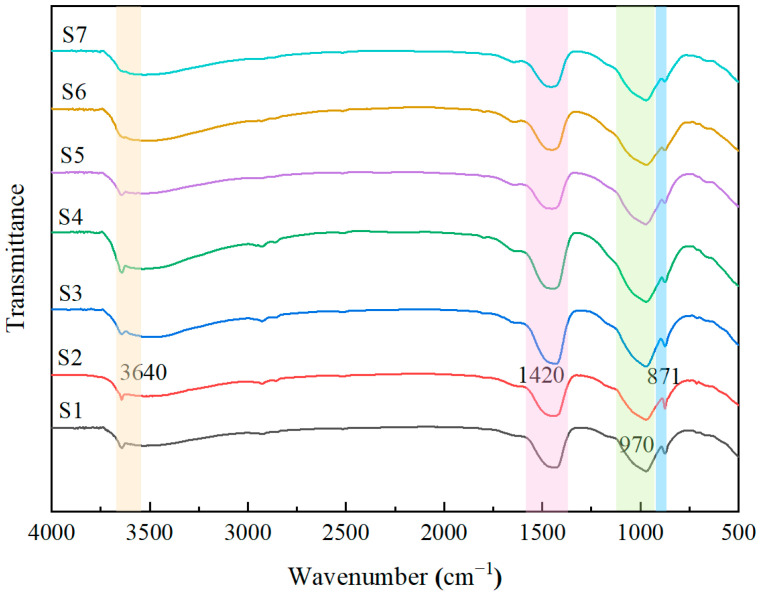
FTIR patterns of AAC samples.

Figure 9 presents the FTIR spectra of AAC samples after autoclaved curing. All samples exhibit four main characteristic peaks. The characteristic peaks at 871 cm^−1^ and 1420 cm^−1^ correspond to the bending vibration and asymmetric stretching vibration of carbon-oxygen (C-O) bonds [26], which are the characteristic absorption peaks of calcium carbonate. Consistent with the results shown in the XRD patterns, the peak intensities at these positions are enhanced in samples S2, S3, and S4, indicating a higher content of calcite in these samples. The characteristic peak around 970 cm^−1^ originates from the asymmetric stretching vibration of silicon-oxygen (Si-O) bonds in the silicate tetrahedral structure of tobermorite [27]. With the increase in the water-to-material ratio, the absorption intensity of this peak gradually increases, indicating the formation of more tobermorite in the system. The characteristic peak appearing near the wavenumber of 3640 cm^−1^ corresponds to the bending vibration of hydroxyl (O-H) bonds, which usually indicates the presence of free water, bound water, and calcium hydroxide in the system [28]. As the water-to-material ratio increases, the intensity of the absorption peak here gradually strengthens.

#### 3.4.3. TG-DTA Analysis

**Figure 10:**
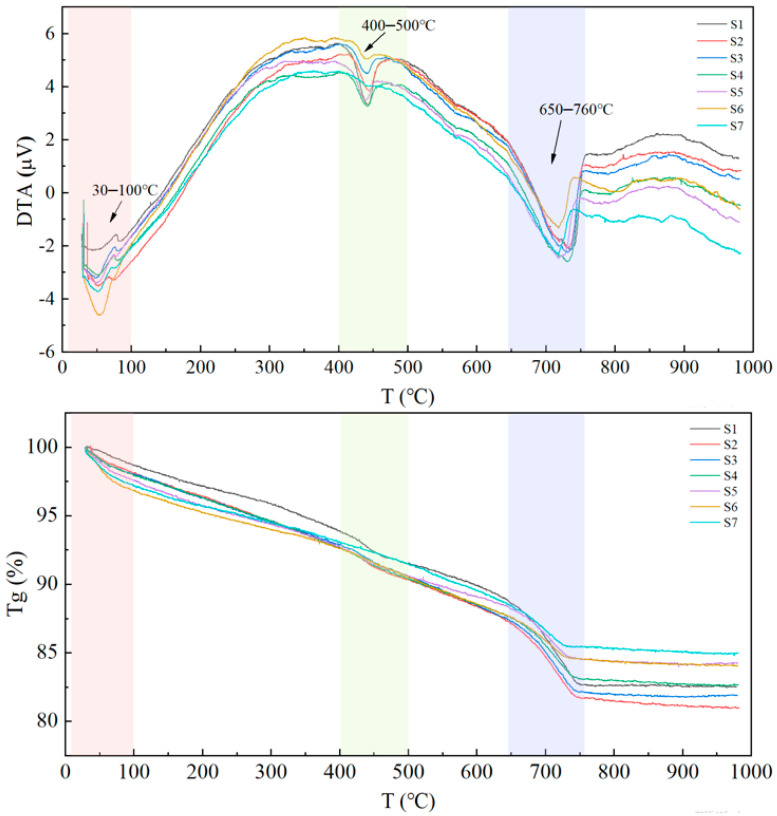
TG-DTA curves of AAC samples.

Figure 10 presents the thermogravimetry (TG) and differential thermal analysis (DTA) curves of the AAC samples. As the ambient temperature rises to 1000 °C, the samples undergo three distinct weight loss stages. Correspondingly, the DTA curves of the samples all exhibit corresponding exothermic peaks, which can be divided into three distinct temperature ranges: 30–100 °C, 400–500 °C, and 650–760 °C.

The first exothermic peak at 30–100 °C and the corresponding TG weight loss are attributed to the removal of free water from the pore structure and hydration products of the autoclaved aerated concrete (AAC) samples [29]. The second decomposition peak at 400–500 °C and the associated TG weight loss are generally related to the dehydration reaction of calcium hydroxide. With the same quicklime dosage, the TG weight losses of samples S1, S2, S3, and S4 are 2.33%, 2.26%, 2.30%, and 2.12%, respectively. A higher content of calcium hydroxide indicates a greater amount of unreacted CaO in the system.

The third decomposition peak at 650–760 °C and the corresponding TG weight loss are mainly associated with the decomposition of calcite and the massive removal of bound water from hydration products [30]. At this stage, the TG weight losses of different samples follow the order: S1 (6.08%) > S2 (5.57%) > S3 (5.29%) > S4 (4.59%) > S5 (3.77%) > S6 (3.12%) > S7 (3.10%).

Under autoclaved curing conditions, the hydrothermal reaction between siliceous and calcareous materials consumes inert quartz and calcium hydroxide (evidenced by a reduced weight loss ratio in the TG-DTA curves), which in turn promotes the formation of well-crystallized tobermorite and xonotlite in the system (supported by the enhanced intensity of characteristic peaks in the XRD patterns). This reaction process is accompanied by a significant intensification of the silicon-oxygen (Si-O) bond vibration absorption peak; with the increase in the water-to-material ratio, the absorption intensity of this characteristic peak rises gradually, which further indicates the formation of more tobermorite in the system.

#### 3.4.4. SEM Analysis

**Figure 11:**
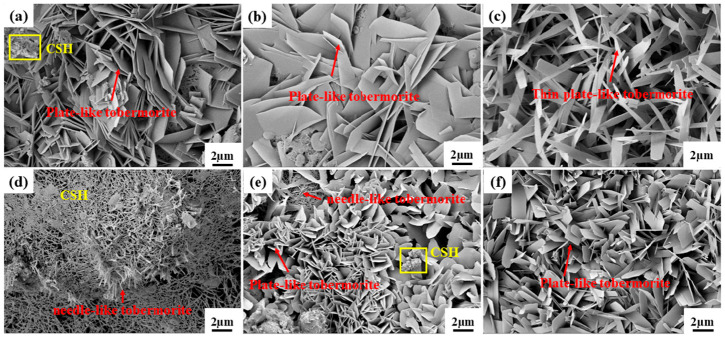
SEM graphs of AAC samples: (**a**) S2 sample, (**b**) S3 sample, (**c**) S4 sample, (**d**) S5 sample, (**e**) S6 sample, (**f**) S7 sample.

Figure 11 shows the microstructure of hydration products in samples S2–S7. As can be seen from the figure, the hydration products of the AAC samples are dominated by tobermorite, with a small amount of C-S-H gel also present. With an increase in the water-to-material ratio, the morphology of tobermorite in the AAC samples gradually transforms from a tightly packed flaky structure to a thin-plate structure, which macroscopically manifests as a decrease in strength.

For Sample S5 (where sediment was replaced by quartz sand), a large amount of C-S-H gel in the sample encapsulates acicular tobermorite. Due to the decrease in the Ca/Si ratio from 0.68 to 0.50, the formation of tobermorite is affected, which is consistent with the XRD results. For AAC samples where slag was replaced by fly ash, the Ca/Si ratio increases to 0.72, and the elevated Ca/Si ratio of the system reaches a level favorable for the formation of plate-like tobermorite [31]. The microstructure is mainly characterized by the co-accumulation of C-S-H gel and plate-like tobermorite; however, the crystal size of tobermorite is smaller than that in AAC samples using slag as the primary raw material.

## 4. Conclusions

(1) Yellow River sediment has been demonstrated as a viable alternative to conventional siliceous raw materials, such as quartz sand, in the production of AAC. Through the optimization of mix proportions and autoclaving conditions, the resulting sediment-based AAC achieved a favorable balance between bulk density and mechanical strength, satisfying the requirements of relevant national standards. This finding overcomes the limitation of low sediment incorporation observed in previous studies and confirms the technical feasibility of utilizing large volumes of sediment in AAC manufacturing.
(2) Within a Portland cement-free alkali-activated system, the alkali activator effectively facilitated the depolymerization of siloxane and aluminate bonds present in the Yellow River sediment. The synergistic interaction between the activated sediment and slag promoted the formation of a dense three-dimensional network structure, predominantly composed of tobermorite and C-S-H gel. This study elucidates the correlation among material composition, pore structure evolution, and macroscopic properties of sediment-based AAC, thereby providing a theoretical foundation for performance regulation in solid waste-based cementitious materials.
(3) The Portland cement-free AAC developed in this research reduces production costs by 15–20% compared to traditional cement-based AAC, demonstrating considerable economic benefits. From an environmental perspective, this technology has the potential to consume 50–80 million tons of Yellow River sediment annually, offering a sustainable solution to the challenge of sediment management in the Yellow River basin.

## Figures and Tables

**Table 1:** Chemical composition of the raw material (%).

Chemical Composition	SiO_2_	CaO	Al_2_O_3_	Fe_2_O_3_	MgO	SO_3_	Na_2_O	K_2_O
Sediment	69.10	8.23	12.51	2.81	1.69	0.05	1.87	3.04
Quicklime	3.18	88.97	1.25	0.73	3.54	1.63	-	0.45
Slag	30.65	41.17	15.31	0.32	7.51	2.28	0.40	0.47
Fly ash	45.04	7.00	20.89	16.76	-	1.79	-	4.12

**Table 2:** The mix proportions of different samples (g).

Sample	Sediment	Quicklime	Slag	Fly Ash	Quartz Sand	TEA	Water
S1	635	200	250	-	-	3	460
S2	635	200	250	-	-	3	470
S3	635	200	250	-	-	3	480
S4	635	200	250	-	-	3	490
S5	500	200	250	-	135	3	470
S6	635	200	125	125	-	3	470
S7	635	200	125	125	-	3	480

**Table 3:** The surface air pore parameters of AAC samples.

Parameter	S1	S2	S3	S4	S5	S6
numPores	223	254	229	234	224	154
numLarge	4	3	11	12	7	1
numMedium	70	81	83	60	71	34
numSmall	149	170	122	162	146	119
areaLarge (%)	33.54	37.17	44.82	64.45	33.56	22.62
areaMedium (%)	58.8	56.48	50.44	31.33	56.78	46.51
areaSmall (%)	7.66	6.34	4.72	4.21	9.67	30.87
MeanDiameter (mm)	0.5161	0.4711	0.5969	0.5532	0.5154	0.3648

## Data Availability

The original contributions presented in the study are included in the article; further inquiries can be directed to the corresponding author.

## Abbreviations

The following abbreviations are used in this manuscript:

AAC	Autoclaved aerated concrete
TEA	Triethanolamine
XRD	X-ray diffractometer
FTIR	Fourier Transform Infrared
TG-DTA	Thermogravimetry–differential thermal analysis
SEM	Scanning electron microscopy
C-S-H	Calcium silicate hydrate
